# Circumscribed interests in adolescents with Autism Spectrum Disorder: A look beyond trains, planes, and clocks

**DOI:** 10.1371/journal.pone.0187414

**Published:** 2017-11-02

**Authors:** Ivy Y. K. Cho, Kristina Jelinkova, Manuela Schuetze, Sarah A. Vinette, Sarah Rahman, Adam McCrimmon, Deborah Dewey, Signe Bray

**Affiliations:** 1 Department of Radiology, Cumming School of Medicine, University of Calgary, Calgary, Alberta, Canada; 2 Child and Adolescent Imaging Research (CAIR) Program, University of Calgary, Calgary, Alberta, Canada; 3 Alberta Children’s Hospital Research Institute, University of Calgary, Calgary, Alberta, Canada; 4 Department of Paediatrics, Cumming School of Medicine, University of Calgary, Calgary, Alberta, Canada; 5 Hotchkiss Brain Institute, University of Calgary, Calgary, Alberta, Canada; 6 Faculty of Medicine, University of Toronto, Toronto, Ontario, Canada; 7 The Ability Hub, Calgary, Alberta, Canada; 8 Department of Psychiatry, Charleston Area Medical Center and West Virginia University Charleston Division, Charleston, West Virginia, United States of America; 9 Werklund School of Education, University of Calgary, Calgary, Alberta, Canada; 10 Department of Community Health Sciences, University of Calgary, Calgary, Alberta, Canada; Swansea University, UNITED KINGDOM

## Abstract

Adolescence is a unique developmental period, characterized by physical and emotional growth and significant maturation of cognitive and social skills. For individuals with Autism Spectrum Disorder (ASD), it is also a vulnerable period as cognitive and social skills can deteriorate. Circumscribed interests (CIs), idiosyncratic areas of intense interest and focus, are a core symptom of ASD that may be associated with social development. Yet, relatively little is known about the expression of CIs in adolescents with ASD. Many studies investigating CIs have used images depicting items of special interest; however, it is not clear how images should be customized for adolescent studies. The goal of this study was to gain insight into the types of images that may be appropriate for studies of CIs in adolescents with ASD. To this end, we used a mixed methods design that included, 1) one-on-one interviews with 10 adolescents (4 with ASD and 6 TD), to identify categories of images that were High Autism Interest (‘HAI’) or High Typically Developing Interest (‘HTD’), and 2) an online survey taken by fifty-three adolescents with ASD (42 male) and 135 typically developing (TD) adolescents (55 male) who rated how much they liked 105 ‘HAI’ and ‘HTD’ images. Although we found a significant interaction between ‘HAI’ and ‘HTD’ categories and diagnosis, neither group significantly preferred one category over the other, and only one individual category ('Celebrities') showed a significant group effect, favored by TD adolescents. Males significantly preferred ‘HAI’ images relative to females, and TD adolescents significantly preferred images with social content relative to adolescents with ASD. Our findings suggest that studies investigating affective or neural responses to CI-related stimuli in adolescents should consider that stereotypical ASD interests (e.g. trains, gadgets) may not accurately represent individual adolescents with ASD, many of whom show interests that overlap with TD adolescents (e.g. video games).

## Introduction

Adolescence is a special developmental period associated with significant changes in physical and emotional growth and the maturation of cognitive and social skills [1,2]. Social development in adolescence is crucial, as the abilities to form successful relationships and show empathy during adolescence are good predictors of successful relationships later in adulthood [3]. Adolescence can be a particularly vulnerable period for those with Autism Spectrum Disorder (ASD) as about one-third of individuals with ASD evidence a deterioration in both cognitive and social skills during this time [4].

Restricted and repetitive behaviours and interests (RRBIs; [5]) are a core symptom of ASD. These repetitive and often non-functional behaviours and interests occur frequently and interfere with daily activities [6]. A specific subtype of RRBIs that are seen in 75–95% [7,8] of children with ASD are circumscribed interests (CIs). CIs are defined as an intense focus on and/or interest in certain objects or topics (e.g. watching the circular movements of a washing machine or memorizing numbers; [7]). Although CIs can allow a child to become very skilled and focused (e.g. becoming efficient with memorizing numbers), the intensity and pervasiveness of CIs can lead to challenges with reciprocal social interaction and communication. Specifically, individuals with ASD may only desire to communicate with those who are also interested in conversing about their specific topics of interest [7,9], and parents and caregivers report that this symptom requires continuous patience and tolerance [6,8].

While several studies have focused on young children and adults [9–11] relatively little is known about the expression of CIs in adolescents with ASD and how they compare to the interests of typically developing (TD) adolescents in content and intensity [12]. CIs are suggested to interact with the development of social behaviour and peer relationships [7,9], and are present in most adolescents with ASD (e.g. 88% in [9]). A greater focus on this symptom is key, as the ability to form friendships in adolescence is predictive of successful adult relationships [3], as for adolescents with ASD the underlying basis of friendships often shifts away from social foundations towards a focus on shared interests [4,13,14].

A challenge with studying CIs is that they are by definition idiosyncratic [15] and can be related to an individual’s gender and age. For example, among preschool children who went on to receive an ASD diagnosis, parent-reported obsession with wheeled toys was strongly predictive of being a boy, while fascination with random objects (e.g. stickers, seashells) or repetitive/obsessive interactions with toys (e.g. teddy bears, figurines) was predictive of being a girl [16]. There also appears to be a developmental component to the content and intensity of CIs [8,17,18]. CIs and associated RRBI symptoms have been generally shown to decline across childhood into adulthood [18]. The content of CIs may also shift with age: in children with ASD CIs often include mechanical and technical items (e.g. trains), while adults with Asperger syndrome show interest in concepts such as art, media, and computers [19,20].

Although historically CIs and associated RRBI symptoms have been considered as behaviors that should be reduced through therapy, it has recently been suggested that in the context of strength-based approaches to therapy, search and exploration of CIs should be encouraged [21]. Indeed, it has been shown that in children ages 4–13 with ASD, incorporating activities that were of interest to the child preferentially decreased social avoidant behaviour [22]. By better understanding the expression of CIs in *adolescents* with ASD, therapies for this relatively underserved group could also benefit.

Despite the prevalence and pervasiveness of CIs among individuals with ASD, this symptom remains relatively understudied compared to the social and communication deficits [7,11]. In laboratory-based studies, images depicting items of interest are often used to investigate behavioral or neural responses to CIs in ASD [23–25]. Much of this work has tested the hypothesis that this symptom is related to hyper-engagement of the brain’s reward circuitry in response to CIs [24]. However, despite a growing interest in understanding the biological basis of CI symptoms in ASD, no studies to our knowledge have used stimuli tailored to adolescents and few have addressed the question of whether behavioral or neural responses to images depicting an in individuals’ interests are exaggerated in individuals with ASD relative to TD controls (though see [26]).

To advance the study of CIs in adolescents with ASD, the present study investigated affective responses using subjective evaluations of images chosen to represent High Autism Interests (‘HAI’) and High Typically Developing Interests (‘HTD’). Images were specifically targeted to adolescents and chosen based on preliminary one-on-one interviews with a small group of adolescents, with and without ASD. Using categories derived from these interviews, image ratings were obtained from a large sample of TD individuals and individuals with ASD using an online survey. Our goals were to gain insight into the types of images appropriate for investigating CIs in adolescents with ASD, and investigate whether individuals with ASD prefer ‘HAI’ compared to ‘HTD’ images compared to TD adolescents.

## Materials and methods

### Recruitment

Participants for both parts of this study (i.e. preliminary interviews and online study) were recruited through existing participant databases at the Alberta Children’s Hospital, as well as community advertisements placed in coffee shops, the University of Calgary campus, community recreation centres and libraries in Calgary, Alberta. Participants with ASD were additionally referred through the Ability Hub at the Child Development Centre and through clinical service providers for adolescents with ASD. The study was approved by the Conjoint Health Research Ethics Board of the University of Calgary. Interviewed participants provided written consent (parents and adolescents over 18) or assent (adolescents under 18). Online study participants provided consent (parents and participants over 18) or assent (under 18) on the first page of the web survey.

### Participants for preliminary interviews (qualitative study)

In order to identify picture categories of interest to adolescents with and without ASD, we conducted a series of one-on-one interviews. Participants in these interviews were ten adolescents including four participants with ASD, three males and one female, aged 14,16,17,18 and six TD adolescents, four males and two females, of which three were aged 14, one aged 15 and two aged 19. Of these participants, one male with ASD and three males and one female in the TD group went on to participate in the online study, described below. It should be noted that measures of social or ASD symptoms and verbal intelligence were only collected from participants who went on to participate in the online survey.

### Preliminary interviews to identify image categories

During the interviews participants were asked to name items and foods they liked and disliked and give examples of hobbies and interests. Based on their responses, we developed categories of interest for TD male and female adolescents: ‘Animals’, ‘Art’, ‘Art Photos’, ‘Buffet’, ‘Celebrities’, ‘Female Sports’, ‘Complex Foods’, ‘Nature Scenes’, ‘Historical Scenes’, ‘Room Designs’, ‘Sports’, ‘Travel’, and ‘Videos’; these categories were considered ‘HTD’. Categories of interest from the interviews with participants with ASD were: ‘Animations’, ‘Simple Foods’, ‘Gadgets’, ‘Lego’, ‘Machines’, ‘Ports’, and ‘Space’; these categories were considered ‘HAI’. Categories such as ‘Gadgets’ and ‘Lego’, categorized to be HAI following the one-to-one interviews, have also previously been found to be items of ‘HAI' in previous studies [11,24,27].

We specifically asked about foods because images of food have been used to investigate neural responses to reinforcement [26,28]. During these preliminary interviews, we found that while TD participants tended to name ‘Complex Foods’ consisting of an amalgam of items (e.g. pizza with different toppings), participants with ASD expressed preferences for images of individual food items, termed here ‘Simple Foods’ (e.g. French fries).

In addition to the ‘HAI’ and ‘HTD’ picture categories, images of money were included as these are often used as reinforcers in studies of reinforcement [24,29]; these were not included in analyses comparing ‘HAI’ to ‘HTD’ images. A total of 21 image categories were identified.

### Picture stimuli

Five pictures corresponding to each of the 21 categories were found using internet search engines (e.g. Google and Pinterest) and were chosen to best represent the picture category without interference of other background items. This resulted in 35 pictures in the ‘HAI’ category, 65 pictures in the ‘HTD’ category, and 5 pictures in the ‘Money’ category for a total of 105 images. All images were scaled to a height of 400 pixels. Individual images, grouped by category, are listed in S1–S3 Tables.

### Online study participants

191 participants, between the ages of 14 and 20 years, and their parents, were recruited to participate in this study. As adolescent development does not abruptly end at 19, our age range extended to 20 years of age (inclusive) in order to facilitate recruiting a larger sample. As part of the survey, parents were asked if the gender of their child was male or female. Three TD participants were excluded due to self-reported diagnoses (two ADHD and one dyslexia). The final sample consisted of 188 participants (mean age = 16.70 ± 1.95 SD years, 97 male), and included 53 participants with ASD (mean age = 16.40 ± 2.01 SD years, 42 male) and 135 TD participants (mean age = 16.82 ± 1.92 SD years, 55 male). Groups did not differ significantly in age (t(186) = -1.35, *p* = 0.18), but did differ in gender distribution (χ^2^ (1) = 21.82, *p* < 0.001). Participant demographics are shown in Table 1.

**Table 1:** Participant demographics.

	ASD	TD	Group difference
Variable	Mean (SD)	Mean (SD)	
**Total N**	53	135	
**Gender (Male/Female)**	(42/11)	(55/80)	χ^2^ (1) = 21.82, *p* < 0.001
**Age**	16.40 (2.01)	16.2 (1.92)	t(186) = -1.35, p = 0.178
**SRS Scores (N = 188)**	75.30 (10.17)	45.51 (6.28)	t(68.16) = 19.90, p < 0.001 *
**SRS RRB Scores (N = 188)**	74.96 (10.64)	46.14 (5.09)	t(18.90) = 61.56, p < 0.001 *
**VI Scores (N = 186)**	22.23 (3.20)	24.42 (1.56)	t(62.06) = -4.77, p < 0.001 *
**# on Medication**	5	0	χ^2^ (1) = 13.08, *p* < 0.001
**# with Comorbidities**	34	0	χ^2^ (1) = 105.72, *p* < 0.001

* indicates a significant group difference at *p* < 0.05.

### Procedure

Participating families completed three online surveys via individualized links that we sent to parents via email. These surveys were generated using SurveyGizmo (Boulder, CO, USA). Participants took the survey on an internet-connected computer of their choice, which was normally at home but may have been at another location. Adolescents were screened to have sufficient verbal ability to complete the surveys on their own, though due to the format of this study we cannot ensure that all participants completed the survey on their own.

Two of these surveys were assigned to the participating adolescent and one was assigned to the parent. Parents were asked to complete the Social Responsiveness Scale, Second Edition (SRS-2), a questionnaire commonly used to assess the presence and extent of social impairment in individuals with ASD [30]. A Total SRS-2 score was calculated as well as a ‘Restricted Interests and Repetitive Behaviour’ subscale score. All raw SRS-2 scores were converted to T-Scores based on age and gender. Adolescents completed (1) 26-questions adapted from the Peabody Picture Vocabulary Test (PPVT-III; [31]) to obtain an estimate of verbal intelligence (VI) and (2) a survey asking them to look at and rate a series of pictures. The adapted PPVT required participants to click on one of four images that depicted a given word (e.g. car). Adapted versions of the PPVT are often used in large studies, including those with ASD participants, where a comprehensive assessment of verbal intelligence, or intelligence more generally, is not practical [32–35]. Here, scores for the 26 questions were summed to calculate an estimated VI score that could range from 0–26. These raw scores were compared between groups; age and gender were included as covariates.

**Fig 1:**
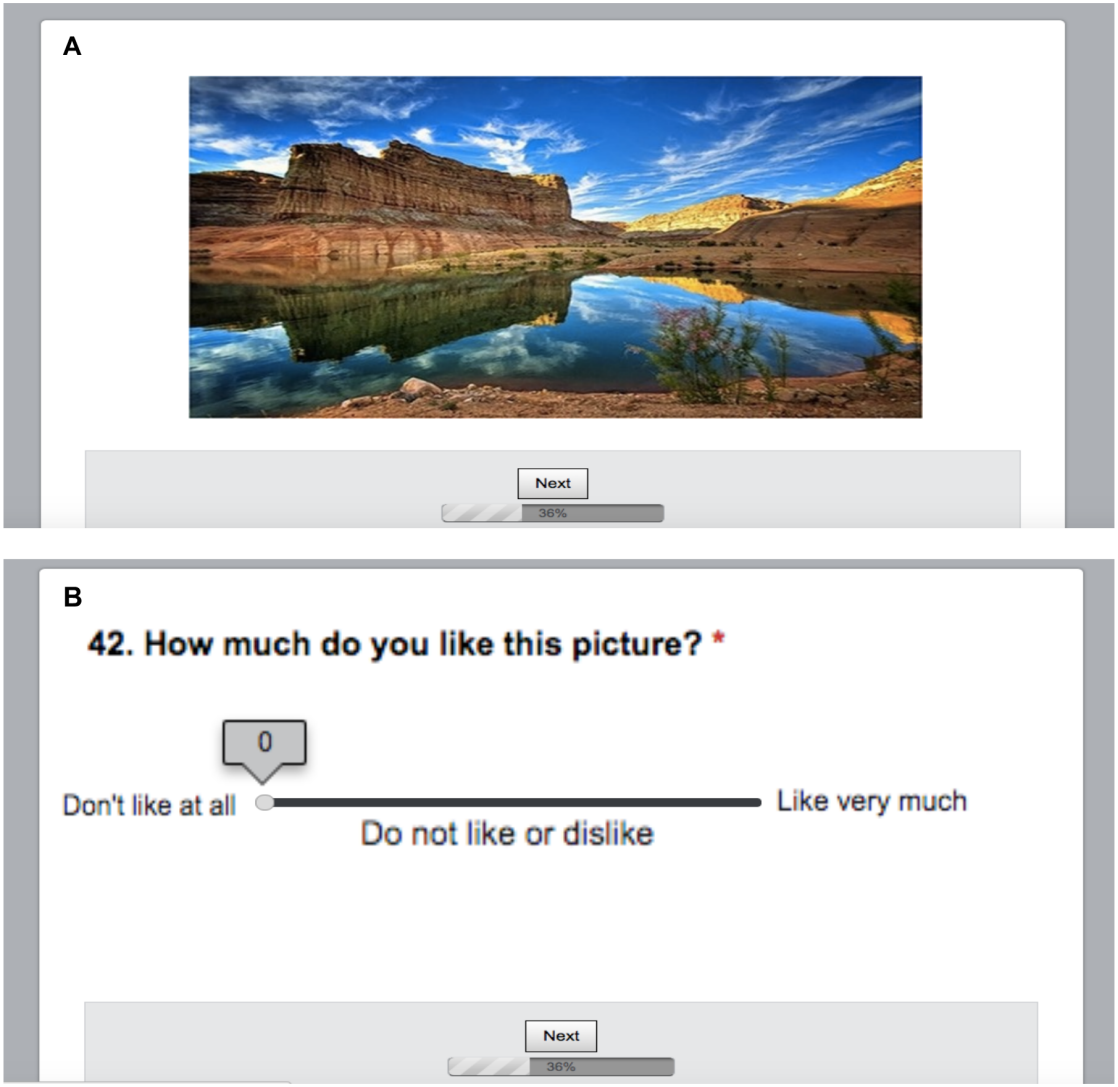
Example question from online survey. (A) Example of an image from the ‘Nature’ category found in the online survey. After viewing the picture for as long as they liked, participants pressed the “Next” button to move on to the next page. (B) After pressing the “Next” button from the previous page, participants were asked how much they liked the image by rating the picture on a scale of 0–7. After rating the image, the participants moved on to the next image by pressing the “Next” button below. Font size has been enlarged from the actual survey for legibility.

For the picture rating survey, participants first saw an instruction screen that stated that they would be presented with a series of pictures, which they could look at the for as long as they liked. After looking at each picture they would be asked to rate on a separate screen how much they liked looking at the picture on a scale of 0 (don’t like it at all) -7 (like it very much). In order to move between screens participants clicked a button with the word “Next” at the bottom of each screen. On the rating screen, participants indicated how much they liked looking at the picture using a sliding bar. Each of the 105 pictures was shown in the same order for every participant; one picture from each category was shown, and the categories were cycled through in the following order: ‘Art’, ‘Art Photos’, ‘Celebrities’, ‘Animations’, ‘Complex Food’, ‘Animals’, ‘Room Designs’, ‘Female Sports’, Buffet’, ‘Gadgets’, ‘Lego’, ‘Machines’, ‘Money’, ‘Videos’, ‘Nature Scenes’, ‘Historical Scenes’, ‘Ports’, ‘Simple Foods’, ‘Space’, ‘Sports’, and ‘Travel’. ‘Break’ screens that featured distracter games and questions were interspersed among the rating trials to keep participants engaged in the survey. An example question is shown in Fig 1.

The time participants spent looking at each screen was also collected. Looking time was measured in milliseconds as the time the page was loaded to the time they clicked “Next” on the picture screen. A limitation of this method is that it also includes time that an individual may have been distracted or looking away rather than looking at the image. In order to mitigate this issue, trials longer than 14.5 seconds were treated as outliers (i.e. likely reflecting distraction), which resulted in removing four trials on average from each participant; 14.5 seconds was selected as histograms showed a sharp drop in viewing times at this level with only 3.8% of responses exceeding this time. One participant with ASD was excluded from this analysis as they had more than 60 trials with viewing times greater than 14.5 seconds.

The picture ratings, and the time spent viewing each picture, were entered into statistical analyses. While the ratings provide a measure of subjective liking, the viewing time could provide insight into how interesting the participants found each picture [36] and how challenging they were to rate. Following completion of the three online surveys participants were emailed a $20 Indigo electronic gift card.

### Statistical analyses

In order to characterize differences in ASD symptoms and VI between ASD and TD groups in our sample, univariate ANCOVAs were used to analyze VI scores as well as SRS-2 Total T-Scores and 'Restricted Interests and Repetitive Behaviors' T-scores including group (ASD, TD) and gender with age as a covariate. We further analyzed raw scores from two SRS-2 questions that specifically relate to CI symptoms.

A repeated measures ANCOVA was used to investigate group differences in ‘liking’ ratings across all 21 individual picture categories, with category, diagnosis and gender included as factors, and age as a covariate. To determine which categories showed group or gender effects, a series of ANCOVA analyses were run for each of the 21 individual categories, with diagnosis and gender as factors and age as a covariate. Inferences were drawn at a significance level of *p* < 0.05, Bonferroni corrected for the number of categories (*p* < 0.05/21 = 0.0023). We also report trend-level effects that survived at *p* < 0.05 uncorrected.

To investigate group differences for ‘HAI’ and ‘HTD’ images overall, we averaged ratings within each of these groupings and analyzed these using a repeated measures ANCOVA, with diagnosis and gender as factors and age as a covariate.

In a follow-up analysis we investigated group differences in ratings based on social content by reclassifying images into ‘Social’ (i.e. images containing people: ‘Celebrities’, ‘Female Sports’, ‘Sports’, and ‘Historical Scenes’) and ‘Non-Social’ (i.e. images that did not contain people: ‘Simple Foods’, ‘Gadgets’, ‘Machines’, ‘Port, ‘Space’, ‘Complex Foods’, ‘Animals’, ‘Art Photos’, ‘Buffet’, ‘Nature Scenes’, ‘Travel’, and ‘Room Designs’) categories. We again used a repeated measures ANCOVA to analyze these data, with diagnosis and gender as factors and age as a covariate. The 'Animations' and ‘Money’ images were excluded from this grouping.

In addition to ratings, the time spent viewing each image was analyzed, as this could provide an objective measure of participants’ interest in the images [36]. After outlier removal, described above, we analyzed viewing time data using the same models as the ratings data.

## Results

### VI and SRS-2 scores

There was a significant effect of diagnosis on VI scores (*F*(1,183) = 19.29, *p* < 0.001) where TD participants had higher scores. A significant interaction was also found between diagnosis and gender (*F*(1,183) = 4.01, *p* = 0.047). This interaction was driven by a smaller difference between TD males and females (mean difference = 0.24; t(131) = 0.87, *p* = 0.38) relative to ASD males and females (mean difference = 1.43; t(51) = -1.33, *p* = 0.19), though neither difference was significant within ASD or TD groups.

As expected, there was a significant effect of diagnosis on SRS-2 Total scores (*F*(1,183) = 492.70, *p* < 0.001); participants with ASD had higher SRS-2 Total scores indicating greater social impairment. There was also a significant effect of gender on SRS-2 Total scores *(F*(1,183) = 6.18, *p* = 0.014); males had higher scores compared to females. A significant interaction between diagnosis and gender was also found (*F*(1,183) = 4.49, *p* = 0.035) where the difference between males and females with ASD was greater than the difference between TD males and females. We also examined SRS-2 'Restricted Interests and Repetitive Behaviors' T-scores and found a significant main effect of diagnosis (*F*(1,183) = 277.26, *p* < 0.001); the mean for participants with ASD (*M* = 4.02, *SD* = 1.56) was greater than TD participants (*M* = 0.47, *SD* = 0.83; scores out of six).

To estimate the extent of CI symptoms within our sample, we examined the summed raw scores for two questions on the SRS-2 that specifically addressed the presence of CI symptoms (‘‘Talks about the same things over and over” and “Has an unusually narrow range of interests”). 68.9% of TD adolescents had a sum of zero (these behaviors are never seen) on these two questions, while only 1.9% of adolescents with ASD had a summed scored zero. However, 66% of adolescents with ASD scored a sum of four or higher (these behaviors are often true or almost always true) while only 0.7% of TD adolescents scored a sum of four. The scores from these two questions were summed and entered into a repeated measures ANCOVA. We found a significant effect of diagnosis (*F*(1,183) = 509.85, *p* < 0.001), where participants with ASD had higher scores. There was also a significant effect of gender (*F*(1,183) = 4.83, *p* = 0.029), where males had higher scores than females.

### ‘Liking’ ratings for individual picture categories

**Fig 2:**
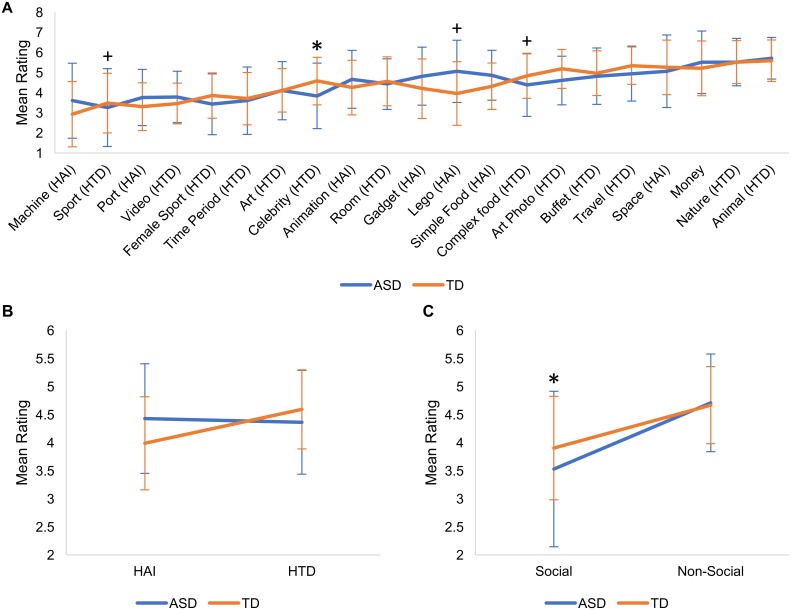
Image “liking” ratings based on diagnosis. (A) Mean picture ratings for individual picture categories based on diagnosis. Only the 'Celebrities' category showed a significant effect of diagnosis, while trend-level effects were seen for ‘Sports’, ‘Lego’ and ‘Complex Foods’ categories. (B) Mean picture ratings for ‘HAI’ and ‘HTD’ super-categories. We found a significant interaction between category and diagnosis, though neither category showed a significant group difference. (C) Mean picture ratings for 'Social' and 'Non-Social' grouped-categories. We found a significant interaction between category and diagnosis, and that 'Social' images were significantly preferred by TD adolescents, relative to adolescents with ASD. “+” = *p* < 0.05 uncorrected; “*" = *p* < 0.05 corrected for multiple comparisons. Error bars depict standard deviation.

**Fig 3:**
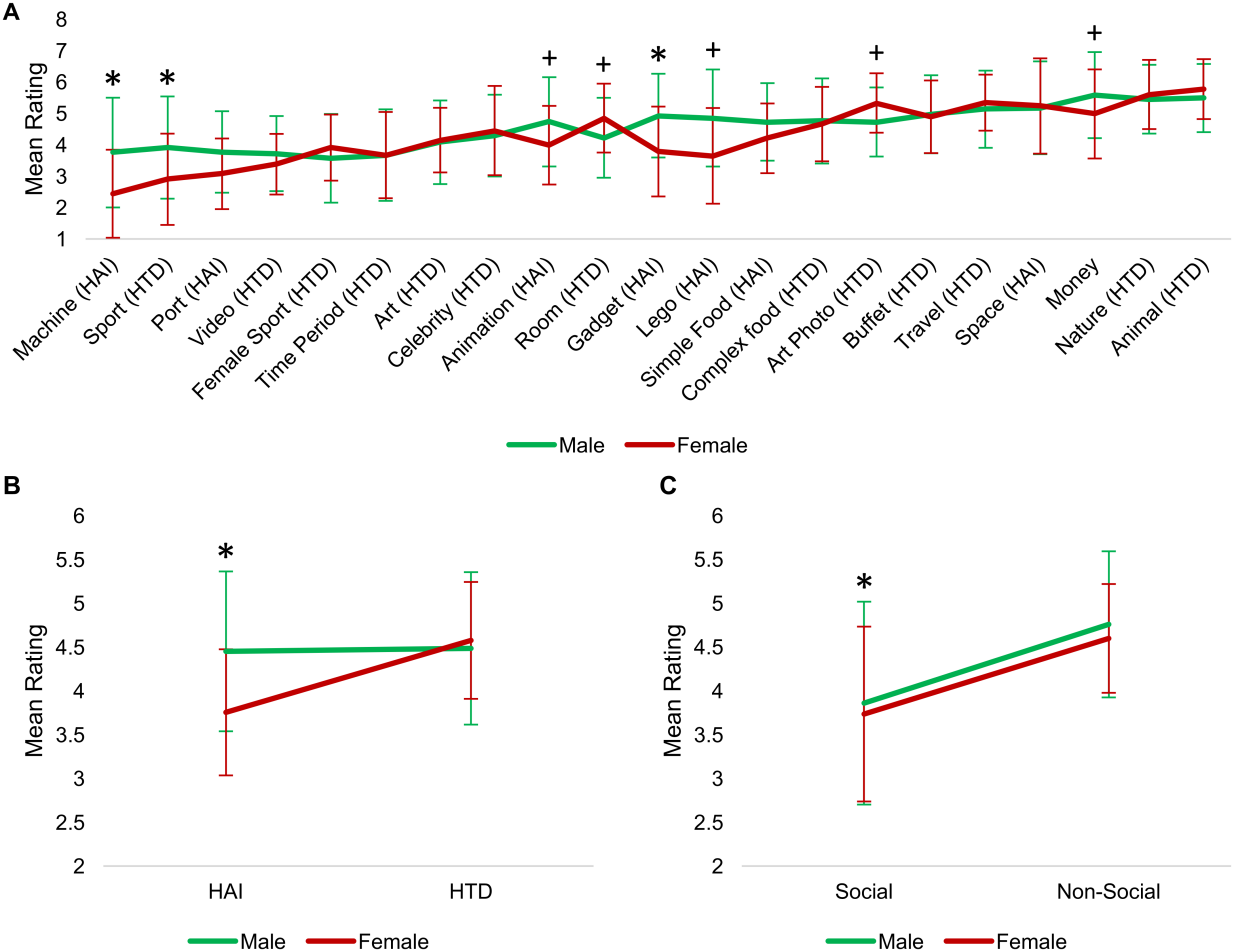
Image “liking” ratings based on gender. (A) Mean picture ratings for individual picture categories based on gender. ‘Machines’, ‘Sports’, and ‘Gadgets’ categories showed a significant effect of gender, while trend-level effects were seen for ‘Animations’, ‘Room Designs’, ‘Lego’, ‘Art Photos’, and ‘Money’ categories. (B) Mean picture ratings for ‘HAI’ and ‘HTD’ super-categories. Males significantly preferred ‘HAI’ images, relative to females. (C) Mean picture ratings for ‘Social’ and ‘Non-Social’ grouped-categories; ‘Social’ images were significantly preferred by males. “+” = *p* < 0.05 uncorrected; “*" = *p* < 0.05 corrected for multiple. Error bars depict standard deviation.

**Fig 4:**
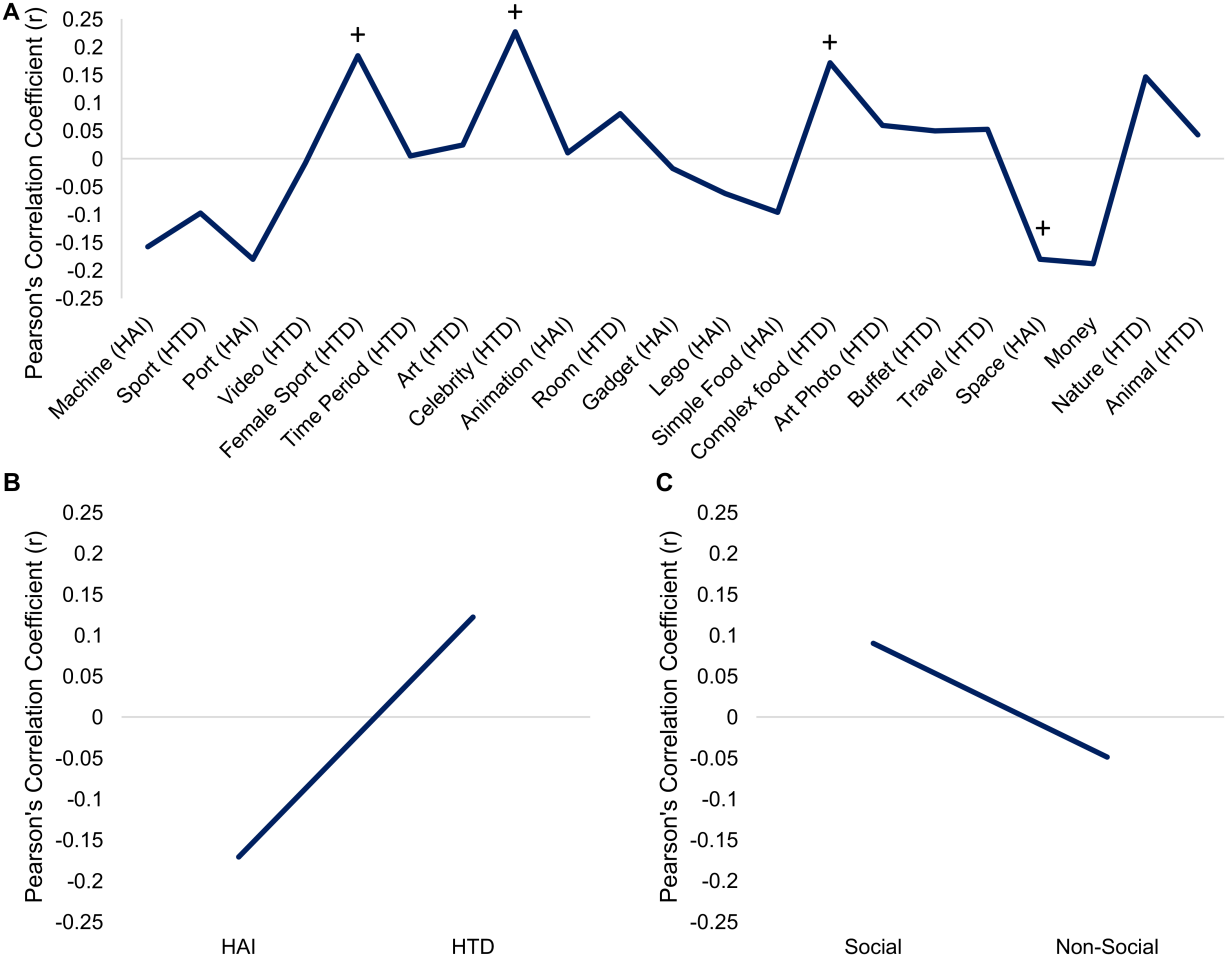
Correlation between “liking” ratings and age. (A) Correlations for individual picture ratings based on age. We found that the picture categories ‘Celebrities’, ‘Female Sports’, ‘Complex Foods’, and ‘Nature Scenes’ showed a positive association with age in ANCOVA models, while ‘Machines’, ‘Ports’, ‘Space’, and ‘Money’ showed a negative association with age. (B) Correlations for ‘HAI’ and ‘HTD’ super-categories based on age. A negative correlation with age was found for ‘HAI’ images. (C) Age correlations for ‘Social’ and ‘Non-Social’ images. “+” = *p* < 0.05 uncorrected; “*" = *p* < 0.05 corrected for multiple comparisons. Error bars depict standard deviation.

Ratings for the individual picture images, grouped by category, are found in S1–S3 Tables. A repeated measures ANCOVA including the 21 picture categories showed a significant main effect of category (*F*(20, 3660) = 4.1, *p* < 0.001), and significant category by diagnosis (*F*(20,3660) = 4.2, *p* < 0.001), category by gender (*F*(20,3660) = 6.7, *p* < 0.001) and category by age (*F*(20,3660) = 3.1, *p* < 0.001) interactions. The three-way interaction was not significant (*F(*20, 3660) = 0.86, *p* = 0.63). Ratings for each category are illustrated in Fig 2A by diagnosis, Fig 3A by gender, and correlations with age are illustrated in Fig 4A.

Looking at individual categories, in order to correct for the number of picture categories (i.e., 21), a Bonferroni correction was used (p < 0.05/21 = 0.0023). Trend-level effects are also reported (i.e., p < 0.05, uncorrected). Only the 'Celebrity' category showed a significant effect of diagnosis (*F*(1,183) = 12.5, *p* = 0.001), with TD participants preferring the pictures in this category. Trend-level (i.e. *p* < 0.05 uncorrected) effects were found for the 'Lego' category (*F*(1,183) = 8.1, *p* = 0.005)—preferred by participants with ASD—and the 'Sports' (*F*(1,183) = 8.9, *p* = 0.003) and ‘Complex Foods’ (*F*(1,183) = 6.4, *p* = 0.012) categories, which were preferred by TD participants.

A significant effect of gender was found for the 'Gadgets' (*F*(1,183) = 13.5, *p* < 0.001), ‘Machines’ (*F*(1,183) = 14.0, *p* < 0.001) and 'Sports' (*F*(1,183) = 23.3, *p* < 0.001) categories, which were favored by males. The 'Money' (*F*(1,183) = 7.5, *p* = 0.007), ‘Lego’ (*F*(1,183) = 8.7, *p* = 0.004), and ‘Animations’ (*F*(1,183) = 6.2, *p* = 0.014) categories showed trend-level effects for gender (i.e. favored by males), and the 'Art Photos' (*F*(1,183) = 8.1, *p* = 0.005) and ‘Room Designs’ (*F*(1,183) = 9.2, *p* = 0.003) categories showed trend-level effects of gender (i.e. favored by females).

A significant of age was found for ‘Celebrities’ (*F*(1,183) = 9.7, *p* = 0.002) and trend level effects for ‘Female Sports’ (*F*(1,183) = 4.7, *p* = 0.03) and ‘Complex Foods’ (*F*(1,183) = 6.4, *p* = 0.012), with older participants preferring pictures in these categories. Younger participants showed a trend-level preference for pictures in the ‘Space’ (*F*(1,183) = 6.7, *p* = 0.011) category.

### ‘Liking’ ratings for ‘HAI’ and ‘HTD’ categories

A repeated measures ANCOVA yielded a significant main effect of category (*F*(1,183) = 7.4, *p* = 0.007) and interactions between category and age (*F*(1,183) = 12.5, *p* = 0.001), category and diagnosis (*F*(1,183) = 15.2, *p* < 0.001), and category and gender (*F*(1,183) = 19.4, *p* < 0.001; Figs 2B, 3B and 4B). The category by diagnosis interaction was driven by a larger difference between ‘HTD’ and ‘HAI’ ratings for the TD group relative to ASD; however, diagnosis was not a significant predictor in either category individually (HAI: *F*(1, 183) = 1.8, *p* = 0.2; HTD: *F*(1, 183) = 2.6, *p* = 0.1). A follow up paired t-test showed that when pooling across participant groups, ‘HTD’ images were liked significantly more than ‘HAI’ (t(1,187) = -7.51, *p <* 0.001). Univariate ANCOVAs accounting for age showed a main effect of gender for the ‘HAI’ images (*F*(1, 183) = 12.7, *p* < 0.001); no significant effects were seen for the ‘HTD’ images. ‘HAI’ images were “liked” more by younger participants, and ‘HTD’ images were “liked” more by older participants, as seen in Fig 4B.

### ‘Liking’ ratings for social and non-social categories

We found a significant main effect of category (*F*(1, 183) = 15.9, *p* < 0.001), and significant interactions between category and diagnosis (*F*(1, 183) = 20.3, *p* < 0.001), and category and age (*F*(1, 183) = 6.05, *p* = 0.015; Figs 2C, 3C and 4C). T-tests showed that both groups rated the ‘Non-Social’ images significantly higher than ‘Social’ (TD: t(134) = -11.45, *p* < 0.001; ASD: t(52) = -7.48, *p* < 0.001). Univariate ANCOVAs showed that ratings in the ‘Social’ category were significantly related to diagnosis (*F*(1,183) = 11.6, *p* = 0.001) and gender (*F*(1,183) = 7.6, *p* = 0.007). As seen in Figs 2C and 3C, ‘Social’ images were preferred by TD participants over participants with ASD and males preferred these images over females. There were no significant effects in the ‘Non-Social’ category. Fig 4C illustrates that ‘Social’ images were “liked” more by older participants, while ‘Non-Social’ images were “liked” more by younger participants.

### Viewing time for individual picture categories

**Fig 5:**
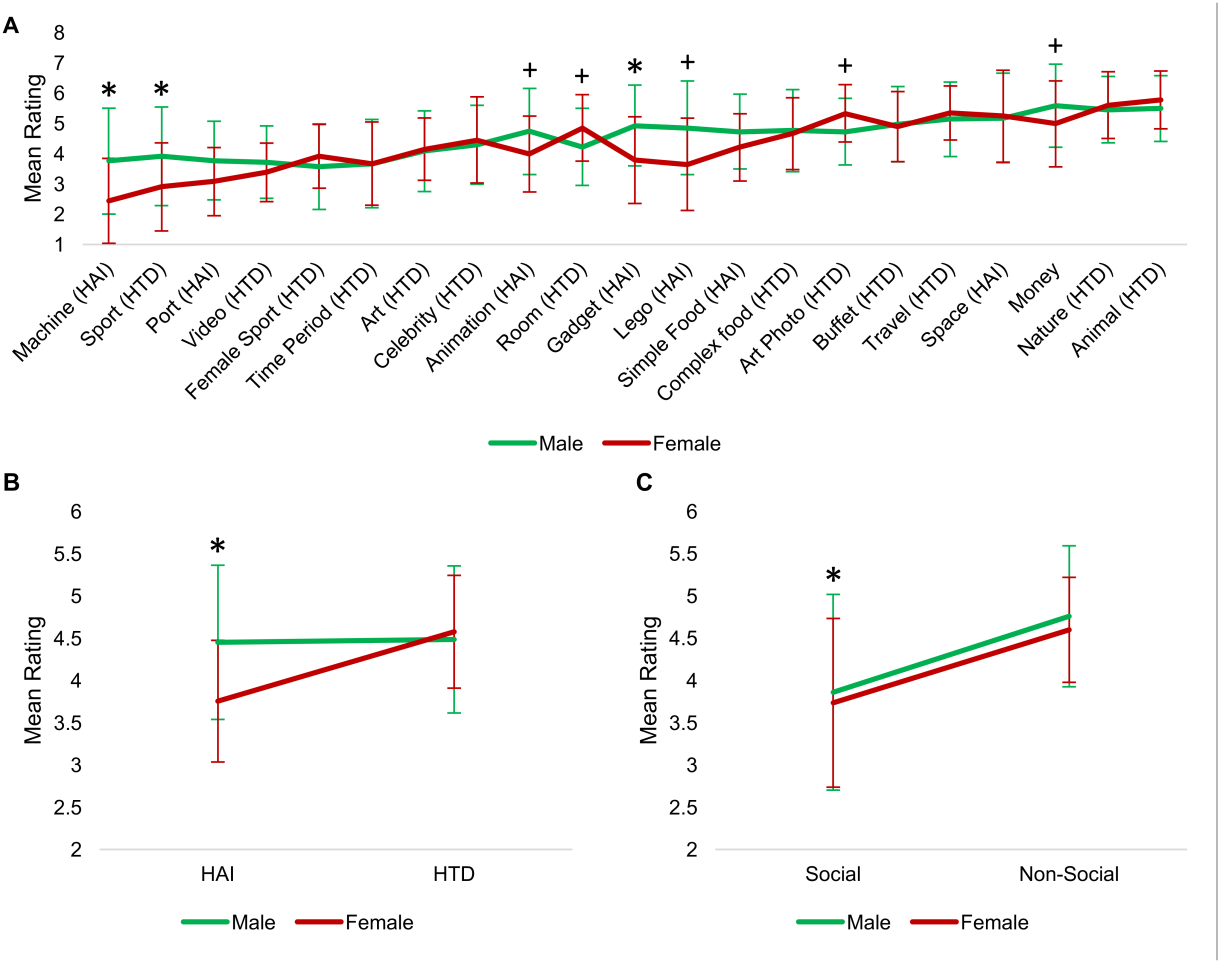
Image viewing times based on diagnosis. (A) Mean picture viewing times for individual picture categories based on diagnosis. Trend-level effects were seen for ‘Money’, ‘Celebrities’, ‘Ports’, and ‘Room Designs’ categories. (B) Mean picture viewing times for ‘HAI’ and ‘HTD’ super-categories. We found a significant effect of diagnosis on ‘HAI’ images. (C) Mean picture viewing times for 'Social' and 'Non-Social' images. 'Non-Social' images were viewed significantly longer by ASD adolescents, relative to TD adolescents. “+” = *p* < 0.05 uncorrected; “*" = *p* < 0.05 corrected for multiple comparisons. Error bars depict standard deviation.

**Fig 6:**
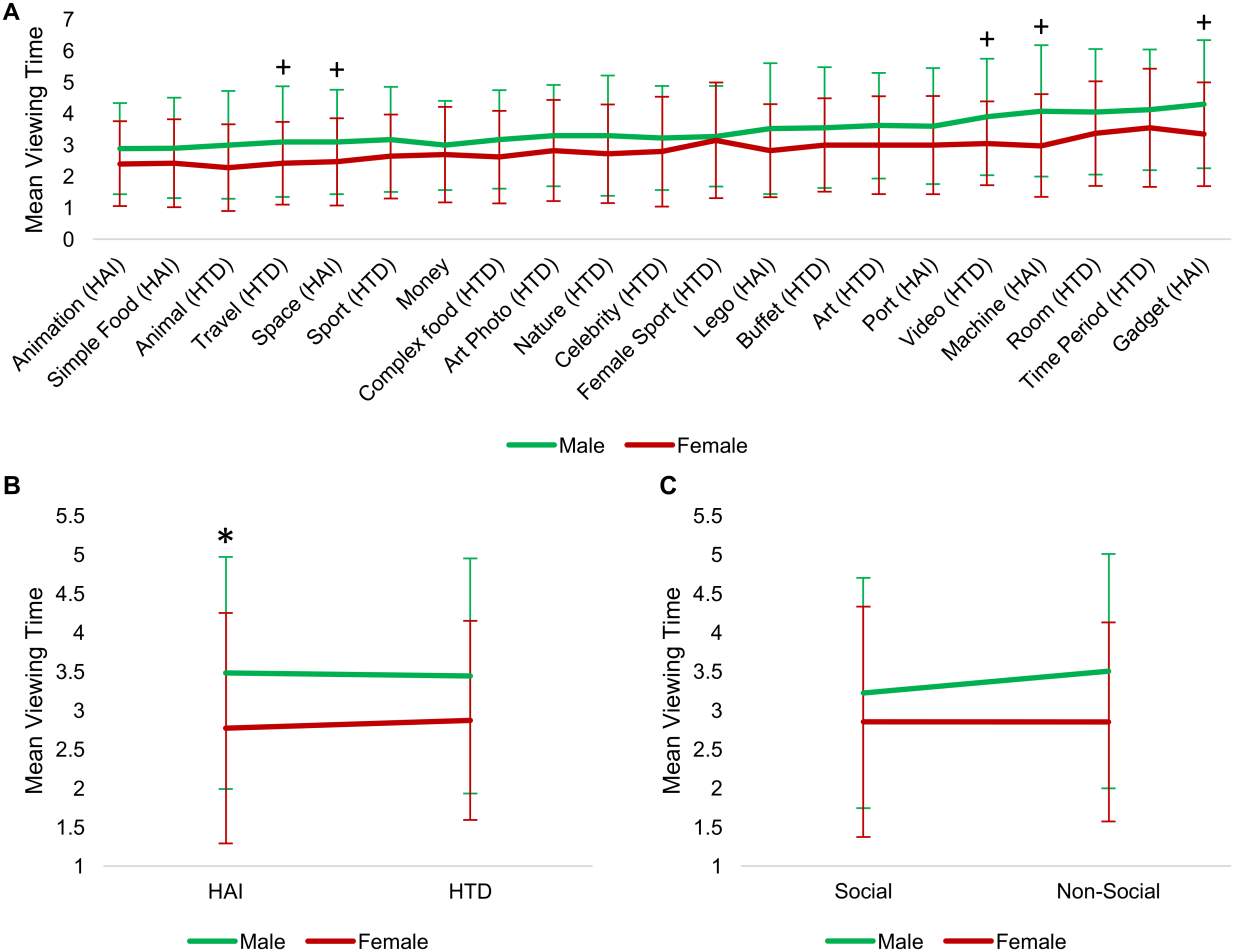
Image viewing times based on gender. (A) Mean picture viewing time for individual picture categories based on gender. Trend-level effects were seen for ‘Travel’, ‘Space’, ‘Videos’, ‘Machines’, and ‘Gadgets’. (B) Mean picture viewing times for ‘HAI’ and ‘HTD' images. ‘HAI’ images were viewed significantly longer by males, relative to females. (C) Mean picture viewing times for ‘Social’ and ‘Non-Social’ images. We found a significant interaction between category and gender, however within each gender category was not significant. “+” = *p* < 0.05 uncorrected; “*" = *p* < 0.05 corrected for multiple comparisons. Error bars depict standard deviation.

**Fig 7:**
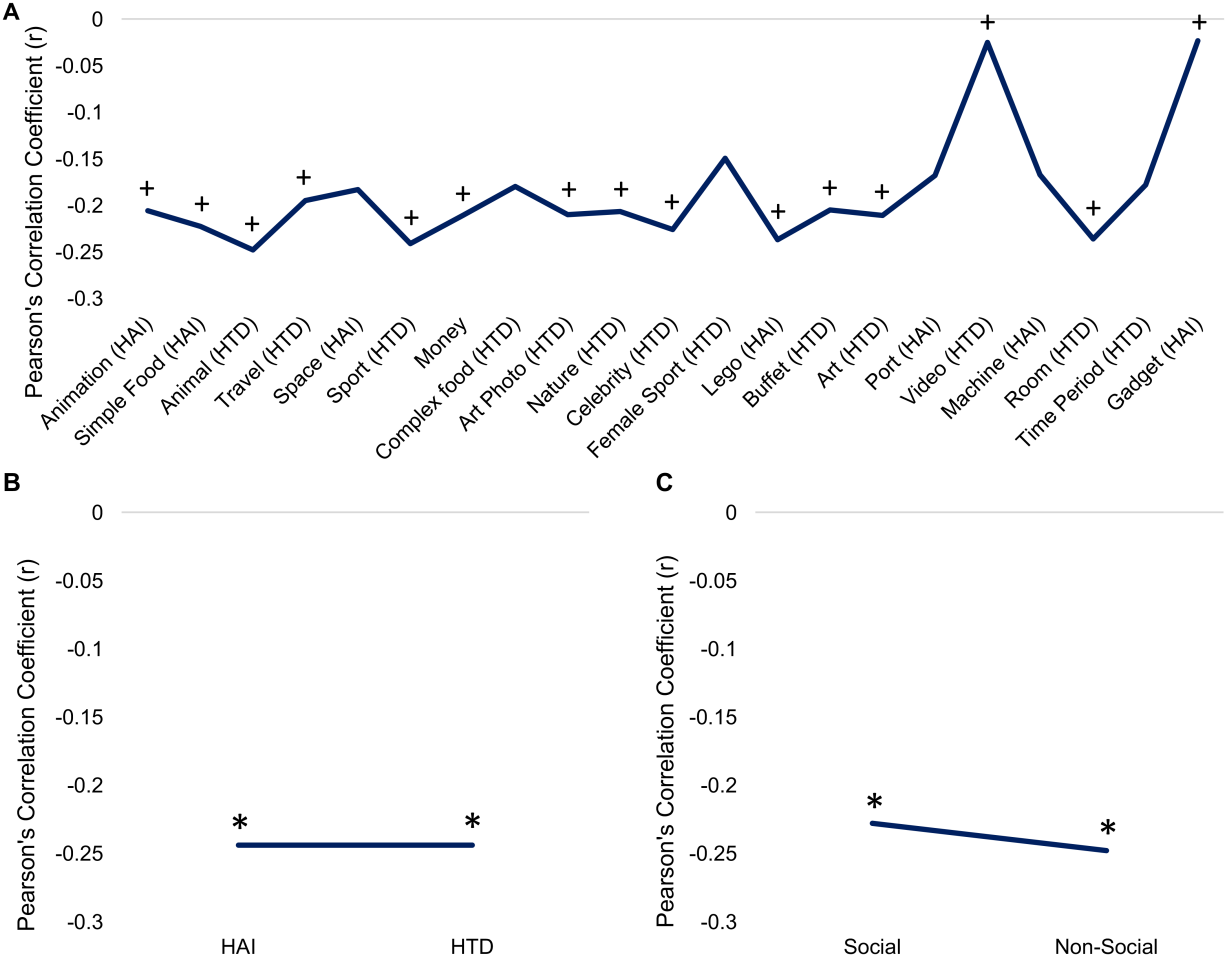
Correlation between viewing times for picture categories and age. (A) Correlations for individual picture viewing times based on age. All categories were found to be viewed longer by younger participants (at significant or trend-level). (B) Age correlations for ‘HAI’ and ‘HTD’ viewing times; younger participants viewed images longer. (C) Age correlations for ‘Social’ and ‘Non-Social’ viewing times; younger participants viewed both ‘Social’ and ‘Non-Social’ images longer than older participants. “+” = *p* < 0.05 uncorrected; “*" = *p* < 0.05 corrected for multiple comparisons. Error bars depict standard deviation.

Viewing times for individual picture categories, grouped by category, are found in S4–S6 Tables. In a repeated measures ANCOVA, we found a significant main effect of age (*F*(1, 182) = 7.9, *p* = 0.005), a trend-level effect of diagnosis (*F*(1, 182) = 3.8, *p* = 0.052), and a significant interaction between category and gender (*F*(20,3640) = 1.6, *p* = 0.045). Means and standard deviations for viewing times are shown in Fig 5A by diagnosis, Fig 6A by gender, and correlations with age are shown in Fig 7A. These results indicate that younger participants viewed pictures for a longer time, there was a tendency for participants with ASD to view images longer and for male participants to view specific categories longer.

For individual picture categories, in order to correct for the number of picture categories (i.e., 21), a Bonferroni correction was used (p < 0.05/21 = 0.0023). Trend-level effects are also reported (i.e., p < 0.05, uncorrected). We found trend-level effects of diagnosis for 'Celebrities' (*F*(1,182) = 7.6, *p* = 0.006), 'Room Designs' (*F*(1,182) = 7.3, *p* = 0.008), 'Money' (*F*(1,182) = 7.5, *p* = 0.007) and 'Ports' (*F*(1,182) = 3.9, *p* = 0.049). All of these categories were viewed longer on average by participants with ASD. Trend-level effects of gender were seen for 'Gadgets' (*F*(1,182) = 5.1, *p* = 0.025), 'Machines' (*F*(1,182) = 4.9, *p* = 0.029), 'Videos' (*F*(1,182) = 5.0, *p* = 0.026), 'Space' (*F*(1,182) = 4.8, *p* = 0.029), and 'Travel' (*F*(1,182) = 5.0, *p* = 0.026) categories. All of these categories were viewed longer by males. Trend-level effects of age were seen for most categories (Fig 7A; *p* values 0.005–0.049); with younger participants viewing images longer. Only the 'Space' category showed a trend-level interaction between diagnosis and gender (*F*(1,182) = 4.1, *p* = 0.045).

### Viewing time for ‘HAI’ and ‘HTD’ categories

Viewing times for ‘HAI’ and ‘HTD’ categories are shown in Figs 5B, 6B and 7B. We found a significant main effect of age (*F*(1,182) = 7.7, *p* = 0.006) and a trend-level effect of diagnosis (*F*(1,182) = 3.8, *p* = 0.052). Univariate ANCOVAs on the ‘HAI’ and ‘HTD’ categories separately showed significant effects of gender (*F*(1,182) = 4.3, *p* = 0.04) and diagnosis (*F*(1,182) = 4.3, *p* = 0.04) only for the ‘HAI’ images. ‘HAI’ images were viewed longer by males and participants with ASD. Both categories showed a main effect of age (‘HAI’: *F*(1,182) = 7.1, *p* = 0.009); ‘HTD’: *F*(1,182) = 7.9, *p* = 0.005)), where younger participants viewed images for longer.

### Viewing time for social vs. non-social categories

Viewing times for these categories are shown in Figs 5C, 6C and 7C. We found a significant main effect of age (*F*(1,182) = 8.1, *p* = 0.005) and a significant interaction between category and gender (*F*(1,182) = 6.5, *p* = 0.011). Univariate ANCOVAs run on the ‘Social’ and ‘Non-Social’ categories separately showed that only the ‘Non-Social’ category had a significant effect of diagnosis (*F*(1,182) = 4.2, *p* = 0.042). The ASD group viewed ‘Non-Social’ images longer than ‘Social’ images (t(51) = -3.43, *p* = 0.001), no difference between these categories were seen in the TD group (t(134) = -1.51, *p* = 0.132). Both 'Social' *(F*(1,182) = 7.77, *p* = 0.006) and 'Non-Social' (*F*(1,182) = 7.7, *p* = 0.006) categories showed a significant effect of age.

### Correlations between picture viewing times and picture ratings

Across groups, picture viewing times and picture ratings were significantly correlated only in the ‘Machines’ (*r* = .280, *p* < 0.001) and ‘Sports’ (*r* = .247, *p* = 0.001) categories.

## Discussion

Despite the prevalence and pervasiveness of CIs in ASD, studies investigating this symptom are limited [12], particularly in adolescence. Behavioural and neuroimaging studies that have investigated CIs often record responses during viewing of images or videos [11,37]. However, most studies to date have not taken into account the specific interests of participants with ASD relative to the hobbies and interests of participants in the control group. Further, there is limited data on how to tailor image stimuli for studies of CIs in adolescents with ASD. Our study was designed to advance the study of CIs in adolescents with ASD by identifying image categories through one-to-one interviews with adolescents with and without ASD. We compared subjective ratings and viewing times between groups, as well as associations with age and gender. In line with previous research, we found that CIs were idiosyncratic [15], as individuals with ASD did not rate images classified as ‘HAI’ significantly higher than ‘HTD’ images. Our results also suggested that individual characteristics (including age and gender) were strongly associated with the ‘liking’ of specific types of images.

In order to understand CIs as a symptom in ASD, it is important to consider how these interests differ in content and intensity from the hobbies and interests of TD individuals. We found that image ratings varied substantially within each group such that when categories were considered individually, only one (‘Celebrities’) was significantly preferred by TD adolescents. We further noted that in the group with ASD there was no significant difference in ratings between images classified as ‘HTD’ or ‘HAI’, suggesting that many ‘typical adolescent’ interests are also enjoyed by individuals with ASD. While previous work has shown that images related to common restricted interests in ASD (e.g. electronics, vehicles) were rated higher and have greater value to individuals with ASD relative to TD [11,27], our study did not replicate these findings and did not show that adolescents with ASD liked ‘HAI’ images more than TD adolescents. However, we did find that ‘HAI’, but not ‘HTD’, images were viewed longer by males and participants with ASD, suggesting that these images may have been more interesting to the ASD group, despite the lack of difference in overt valuations. Overall, these findings suggest that care should be taken to ensure that if images related to interests of TD individuals are used as ‘control images’ that they do not overlap with items of interest to individuals in the ASD sample.

We found that gender was a significant predictor of ratings for several categories, and males “liked” ‘HAI’ images significantly more than females, while individuals with ASD did not like them more than ‘HTD’ images. A recent study showed that 'HAI' images were rated higher by both males and individuals with ASD [11]. However, in contrast to a previous study that found that image categories such as ‘Trains’ and ‘Vehicles’ were rated higher in arousal by males with and without ASD compared to females [11], we found that several ‘HTD’ image categories including ‘Sports’ were preferred by male participants. These results suggest that an emphasis on CIs in ASD as male-typical [11,38] may be limiting in that it does not account for the range of interests of TD males. However, we also note that a recent study of parents of children with ASD aged 5–18 found that male and female children with ASD are equally likely to report 'special interests' but that the content of these interests tend to separate along 'traditional gender lines' [39]. Together with our work, these findings suggest that the interaction between diagnosis and gender is important for understanding the presentation of CIs in ASD.

Images classified as ‘Social’ were preferred by TD adolescents, relative to adolescents with ASD, consistent with previous literature [11]. Even from preschool years, children with ASD show a reduced interest towards social stimuli (e.g. pictures of children) [40]. This reduced interest in social stimuli has also been seen in adolescents, where a reduction in viewing times for social stimuli compared to non-social stimuli has been reported [41]. Interestingly, we found no group differences in ratings of “liking” for images classified as ‘Non-Social’; however, these ‘Non-Social’ were viewed longer by participants with ASD, relative to TD. The difference in viewing times for 'Social' images was not significantly different between groups. If we interpret longer viewing time as an indication of increased arousal (as suggested by [36]), our results differ from a previous study which did not find significant differences between adults with and without ASD in arousal ratings for ‘Non-Social’ images [11]. However, our findings are similar to a study showing that adolescents with ASD fixated on non-social stimuli longer than TD individuals [42]. Interestingly, in our preliminary interviews individuals with ASD suggested fewer categories that involved people yet they did provide examples of animated characters, which have a social element.

Both ratings and viewing times were affected by participant age, and as such our results add to the literature regarding the developmental component of CIs. Only one significant age effect was seen for category ratings, the ‘Celebrities’ category, which was preferred by older participants. Overall ‘HAI’ images were “liked” more by younger participants, and ‘HTD’ images by older participants, suggesting that ‘HAI’ items may reflect more immature interests. A significant age effect was seen for viewing times of ‘HAI’ and ‘HTD’, and ‘Social’, and ‘Non-Social’ images, where younger participants viewed images in these categories longer. This may suggest that attention to images of interest is stronger at a younger age in adolescents with and without ASD. Previous literature has suggested that RRBIs are stronger in intensity at a younger age [18,43], but research on CIs has reported inconsistent findings, suggesting that they either increase or decrease in intensity/pervasiveness with age [8,18]. Discrepancies in previous work could be related to the use of different measurements of CI symptoms: South et al. (2005) used the Yale Special Interests scale to measure CIs, while Shattuck et al. (2007) used two questions related to CIs from the Autism Diagnostic Interview—Revised measure. Here, we did not find any suggestion of an age effect in CIs or Restricted Interest and Repetitive Behavior symptoms, as measured by the SRS-2.

Viewing time data showed a complex pattern with a trend towards pictures being viewed overall longer by participants with ASD, which makes viewing times for individual picture categories challenging to interpret. Difficulties in navigating through the survey for adolescents with ASD may have contributed to longer viewing times compared to TD adolescents. This difference reached trend-level for specific categories, including 'Celebrities' which was rated significantly higher by TD adolescents. Despite this, the adolescents with ASD viewed 'Non-Social' images overall longer than they viewed 'Social' images, and viewed 'HAI' but not 'HTD' images longer than TD participants. As viewing times have been linked to arousal rather than valence [36], our findings may reflect a curiosity for certain items independent of how much they are 'liked'. However, given the limitation that our viewing time measure is less precise than eye-tracking and we cannot control for time spent looking away from the screen, this suggestion is highly speculative.

Pictures of money were included as these have frequently been used in neuroimaging studies of reward learning, including in studies of ASD [44,45]. We found no significant group effect of ratings for monetary images and only a trend level effect of viewing times for monetary images. A previous study investigating monetary and social rewards in ASD showed that both TD individuals, and individuals with ASD, place greater value on images depicting greater monetary value [27]. With no significant group differences for ratings or viewing times, our findings provide support for the use of images of money as plausible incentives in studies of adolescents with ASD, similar to TD adolescents.

Although using images tailored to an adolescent group and a relatively large sample were strengths of this study, there are several limitations. First, the complexity of images was not controlled for; hence, ratings and to a greater extent, viewing times, may have been influenced by image complexity. While there were only seven picture categories in our ‘HAI’ category, there were nearly twice as many in our ‘HTD’ category (i.e. 13). This same imbalance was seen between the 'Social' and 'Non-Social' categories, with four picture categories in 'Social' and twelve in the 'Non-Social' category. This imbalance may have led to less accurate estimates of ratings and viewing times for under-represented categories. The order of picture presentation was not random, which could have led to expectations and order effects. Fatigue towards the end of the survey may have resulted in participants viewing pictures for shorter lengths of time in order to finish more quickly. As this was an online study, we did not confirm ASD diagnosis through the administration of a confirmatory assessment such as the Autism Diagnostic Observation Schedule, Second Edition (ADOS-2; [46]). However, we note that the SRS-2 scores showed robust differences between groups on social symptom severity. In addition, using the SRS to describe CIs is limited as only two questions were available to measure this symptom and as such is likely insufficient to capture the full expression of the symptom. In the future, other scales, such as the Yale Special interests Scale (as used in [8]), could be used to measure CIs as they provide a greater variety of questions to measure symptomology. The gender distribution between the ASD and TD groups was uneven: the TD sample included a higher proportion of females (59.26%) relative to the ASD sample (20.75%). Here, participants with ASD had lower estimated VI scores than the TD participants and differences in ratings and viewing times may have been due to lower verbal and/or overall intellectual functioning. However, the use of a subset of PPVT-III questions is not a validated assessment of VI and moreover may have led to underestimating intelligence in participants with ASD, because individuals with ASD often score higher on non-verbal IQ compared to verbal IQ assessments [47]. Another limitation to note is that this study measured “liking” of images rather than allowing participants to interact with real objects, and therefore may not be an accurate reflection of interest in the items presented. However, images are often used by necessity in behavioral, eye-tracking and neuroimaging studies, and do engage affective responses in the brain and behavior [23–25,48]. Finally, image categories were chosen based on interviews with a small group of adolescents with and without ASD and therefore cannot represent the full range of interests seen in adolescents with and without ASD.

## Conclusions

Our findings indicated that adolescents with ASD do not display many stereotypical ASD interests (e.g. clocks, trains) among their special interests. Instead they shared many ‘typical adolescent’ interests. Moreover, “liking” of images depicting adolescent interests varied substantially depending on the individual. We further found that the tendency to “like” certain image categories was associated with diagnosis, age and gender. To our knowledge, this is the first study to investigate affective ratings of CIs in adolescents with ASD using ‘HTD’ picture categories as comparison images, rather than social or low-autism-interest images. This is important as it could provide guidance for developing image sets tailored to interests of individuals both with and without ASD. This, in turn, can facilitate studying how behavioral and neural responses to CIs in ASD may differ from the responses of TD individuals to their own hobbies and interests. Insights gained from studying CIs may help to improve therapies for individuals with ASD [9,49] and develop strategies to support the positive aspects [50,51] and mitigate the negative aspects of this symptom [52].

## Supporting information

S1 Table Picture ratings for High TD Interest (HTD) images. Possible scores ranged from 1–7.
(PDF)

S2 Table Picture ratings for High Autism Interest (HAI) images. Possible scores ranged from 1–7.
(PDF)

S3 Table Ratings for money images. Possible scores ranged from 1–7.
(PDF)

S4 Table Viewing times (in seconds) for High TD Interest (HTD) images. (PDF)

S5 Table Viewing times (in seconds) for High Autism Interest (HAI) images. (PDF)

S6 Table Viewing times (in seconds) for money images. (PDF)
