# Supplementary material for: Circumscribed interests in adolescents with Autism Spectrum Disorder: A look beyond trains, planes, and clocks
Source: PLoS One. 2017 Nov 2;12(11):e0187414. doi: 10.1371/journal.pone.0187414 (PMC5667845; doi:10.1371/journal.pone.0187414)
Supplement: S1 Table — Possible scores ranged from 1–7. (PDF) [file pone.0187414.s001.pdf]

S1 Table. Picture ratings for High TD Interest (HTD) images. Possible scores ranged from 1-7.

Category	Image	ASD Males	ASD Females	TD Males	TD Females
Animals	Bunny	5.20 (1.53)	5.73 (1.31)	5.25 (1.55)	5.43 (1.42)
	Kitten	5.82 (1.57)	5.73 (1.49)	5.43 (1.34)	5.81 (1.29)
	Knut (Polar Bear)	5.61 (1.38)	6.09 (1.00)	5.14 (1.54)	5.71 (1.16)
	Puppy	6.00 (1.08)	6.09 (1.51)	5.69 (1.42)	5.93 (1.12)
	Teacup Pig	5.59 (1.35)	5.59 (1.32)	5.33 (1.67)	5.78 (1.49)
Art	Basquiat	2.35 (2.12)	3.23 (2.59)	2.89 (2.03)	2.63 (1.90)
	Modern Sculpture 1	4.34 (1.96)	4.45 (1.97)	4.24 (1.66)	4.28 (1.45)
	Bruce	4.41 (1.71)	4.36 (1.79)	3.84 (1.89)	3.88 (1.72)
	Mona Lisa	4.44 (1.96)	3.82 (2.24)	4.42 (1.68)	4.66 (1.42)
	Starry Night	4.95 (1.82)	4.82 (1.78)	4.92 (1.75)	5.28 (1.47)
Art Photos	Bicycle	4.92 (1.75)	5.73 (1.46)	5.46 (1.13)	5.81 (1.08)
	Birds	4.20 (2.14)	5.64 (1.66)	4.44 (1.77)	4.90 (1.61)
	Bridge	4.13 (1.77)	4.50 (1.69)	5.04 (1.41)	5.14 (1.58)
	Hot Air Balloon	4.76 (1.75)	5.50 (1.61)	5.18 (1.48)	5.46 (1.45)
	Ride	4.41 (1.85)	4.36 (1.89)	4.53 (1.59)	5.58 (1.13)
Buffet	Snacks	4.61 (2.07)	4.27 (1.63)	5.11 (1.47)	5.22 (1.48)
	Candy Party	5.20 (2.07)	4.91 (1.46)	4.74 (1.65)	4.93 (1.52)
	Christmas	5.01 (1.72)	4.95 (1.69)	5.08 (1.49)	5.01 (1.34)
	Lunch	4.49 (2.18)	4.50 (2.30)	4.92 (1.38)	4.71 (1.50)
	Pizza Table	5.01 (1.81)	3.82 (2.05)	5.10 (1.53)	5.13 (1.44)
Celebrities	Angelina Jolie	4.45 (1.85)	3.68 (1.68)	4.88 (1.57)	4.11 (1.45)
	Channing Tatum	3.60 (2.14)	2.95 (1.82)	3.77 (1.78)	4.72 (1.85)
	Blake Lively	4.13 (1.99)	3.41 (2.22)	4.81 (1.59)	4.34 (1.62)
	Joseph Gordon Levitt	3.33 (2.05)	3.59 (1.80)	4.14 (1.51)	4.98 (1.75)
	Emma Watson	4.39 (2.03)	3.68 (2.64)	4.98 (1.54)	4.79 (1.49)
Female Sports	Equestrian	3.42 (1.93)	4.05 (1.93)	3.89 (1.65)	3.99 (1.51)
	Ringette	3.45 (2.17)	1.95 (1.85)	3.60 (1.84)	2.73 (1.48)

	Figure Skating	3.39 (2.31)	3.09 (2.26)	3.70 (1.77)	4.40 (1.46)
	Gym	3.40 (2.05)	4.45 (2.04)	3.63 (1.77)	4.30 (1.47)
	Synchronized Swimming	3.60 (2.07)	3.86 (2.13)	3.98 (1.67)	4.32 (1.64)
Complex Foods	Bibimbap	3.38 (2.37)	3.95 (2.25)	4.79 (1.68)	4.96 (1.73)
	Pizza	5.34 (1.87)	4.00 (2.59)	5.30 (1.68)	5.13 (1.63)
	Salad	3.65 (2.16)	4.18 (2.40)	4.37 (1.70)	4.56 (1.32)
	Steak	4.99 (2.04)	5.14 (1.90)	5.21 (1.58)	4.44 (2.07)
	Tacos	4.89 (1.96)	3.36 (2.53)	5.15 (1.40)	4.58 (1.72)
Nature Scenes	Beach	5.56 (1.23)	5.68 (1.06)	5.64 (1.32)	5.57 (1.49)
	Canyon	5.48 (1.28)	5.86 (1.34)	5.53 (1.40)	5.64 (1.37)
	Plains	5.20 (1.62)	5.64 (1.61)	4.93 (1.41)	5.33 (1.43)
	Mountain	5.56 (1.54)	5.77 (1.15)	5.49 (1.37)	5.84 (1.11)
	Forest	5.47 (1.62)	5.55 (1.44)	5.39 (1.44)	5.62 (1.38)
Historical Scenes	Gladiator	4.02 (1.88)	2.82 (1.68)	4.31 (1.74)	3.16 (1.43)
	Marie Antoinette	3.33 (2.14)	4.00 (2.42)	3.35 (1.68)	4.20 (1.55)
	King Arthur	3.94 (1.97)	3.73 (1.77)	4.14 (1.63)	3.55 (1.62)
	Pride and Prejudice	3.48 (2.02)	3.55 (2.30)	3.61 (1.48)	4.06 (1.83)
	Young Victoria	3.30 (1.90)	3.91 (2.03)	3.34 (1.84)	3.71 (1.76)
Room Designs	Swimming Room	5.89 (1.13)	6.18 (1.08)	5.90 (1.26)	6.11 (1.43)
	Jeans Room	4.34 (1.84)	5.18 (1.40)	4.41 (1.73)	4.92 (1.53)
	Paris Themed Room	3.38 (2.23)	3.73 (2.37)	3.14 (1.84)	4.08 (1.84)
	Purple Themed Boy Room	4.44 (2.18)	4.59 (2.03)	4.30 (1.77)	4.01 (1.98)
	Purple Themed Girl Room	3.31 (2.09)	5.73 (1.13)	3.55 (1.81)	4.96 (1.48)
Sports	Baseball	3.63 (2.16)	1.55 (1.51)	3.76 (1.91)	2.72 (1.57)
	Basketball	3.45 (2.19)	2.05 (2.26)	4.24 (1.95)	3.13 (1.71)
	Football	3.64 (2.36)	1.23 (1.46)	4.27 (1.82)	3.06 (1.74)

	Hockey	4.16 (2.42)	1.95 (2.14)	4.26 (2.00)	3.02 (1.70)
	Soccer	3.34 (2.18)	2.14 (2.41)	4.05 (2.15)	3.37 (1.76)
Travel	Asia	5.06 (1.68)	4.77 (2.05)	5.18 (1.25)	5.31 (1.37)
	Italy	4.85 (1.84)	5.05 (1.89)	5.42 (1.31)	5.84 (1.20)
	Pyramids	4.50 (1.80)	4.18 (1.62)	5.14 (1.33)	4.67 (1.59)
	Sydney	5.32 (1.57)	5.68 (1.40)	5.30 (1.32)	5.51 (1.33)
	Eiffel Tower	4.92 (1.70)	5.32 (1.08)	5.25 (1.43)	5.62 (1.08)
Videos	Crash My Party	4.65 (1.71)	4.59 (1.30)	4.37 (1.68)	3.66 (1.87)
	Gangnam Style	3.74 (2.67)	4.14 (1.83)	3.43 (2.10)	2.92 (1.79)
	Tres Semana	3.34 (1.66)	2.45 (1.54)	3.03 (1.43)	2.87 (1.48)
	Thriller	4.23 (2.41)	3.55 (2.32)	4.53 (1.54)	4.12 (1.58)
	Radioactive	2.98 (2.01)	4.45 (2.39)	3.33 (2.00)	3.13 (1.92)
