# Supplementary material for: Circumscribed interests in adolescents with Autism Spectrum Disorder: A look beyond trains, planes, and clocks
Source: PLoS One. 2017 Nov 2;12(11):e0187414. doi: 10.1371/journal.pone.0187414 (PMC5667845; doi:10.1371/journal.pone.0187414)
Supplement: S2 Table — Possible scores ranged from 1–7. (PDF) [file pone.0187414.s002.pdf]

**S2 Table. Picture ratings for High Autism Interest (HAI) images.** Possible scores ranged from 1-7.

| Category | Images | ASD Males | ASD Females | TD Males | TD Females |
|---|---|---|---|---|---|
| Animations | Bugs Bunny | 5.03 (1.92) | 4.82 (1.68) | 4.97 (1.56) | 4.51 (1.40) |
| | Buzz Lightyear | 4.01 (2.30) | 4.09 (1.38) | 4.19 (2.04) | 3.79 (1.62) |
| | Homer Simpson | 4.77 (2.13) | 3.50 (2.26) | 4.81 (2.00) | 3.31 (1.85) |
| | Pikachu | 4.81 (2.06) | 5.50 (2.09) | 4.58 (1.90) | 3.99 (2.04) |
| | Scooby Doo | 4.85 (1.93) | 3.77 (2.28) | 4.99 (1.89) | 4.25 (1.66) |
| | | | | | |
| Simple Foods | Cereal | 3.65 (1.94) | 3.91 (1.63) | 3.96 (1.48) | 3.68 (1.64) |
| | Chicken | 4.75 (2.22) | 4.68 (1.65) | 4.27 (1.97) | 3.27 (2.10) |
| | Fries | 5.34 (1.46) | 4.64 (1.82) | 4.79 (1.63) | 4.53 (1.80) |
| | Ice Cream | 5.49 (1.36) | 4.91 (2.06) | 5.14 (1.45) | 5.00 (1.57) |
| | Toast | 5.18 (1.68) | 4.64 (1.83) | 4.52 (1.47) | 4.44 (1.53) |
| | | | | | |
| Gadgets | Chair | 5.09 (1.78) | 4.50 (1.30) | 5.00 (1.68) | 4.43 (1.83) |
| | Car | 4.92 (2.08) | 3.32 (2.42) | 5.33 (1.69) | 3.83 (1.94) |
| | Electronic Bike | 4.36 (2.14) | 3.68 (1.55) | 4.32 (1.77) | 3.38 (1.94) |
| | Headphones | 5.47 (1.72) | 4.55 (2.07) | 5.08 (1.42) | 3.82 (1.76) |
| | Solar Panel Sunglasses | 5.15 (1.66) | 3.95 (1.71) | 4.69 (1.75) | 3.72 (1.72) |
| | | | | | |
| Lego | Brick Avengers | 5.19 (1.95) | 4.95 (1.69) | 4.61 (1.58) | 3.66 (1.50) |
| | Art | 5.02 (1.96) | 4.77 (1.33) | 4.58 (1.89) | 3.49 (1.96) |
| | Polar Bear | 4.93 (1.77) | 4.77 (2.15) | 4.64 (1.74) | 3.99 (1.77) |
| | R2D2 | 5.41 (1.73) | 3.91 (1.71) | 4.63 (1.83) | 3.60 (1.88) |
| | Ship | 5.18 (1.80) | 4.41 (1.92) | 4.68 (1.73) | 3.35 (1.76) |
| | | | | | |
| Machines | Bonsack | 4.38 (1.87) | 3.00 (2.01) | 4.43 (1.67) | 2.98 (1.61) |
| | MD1 | 3.61 (2.00) | 2.82(1.95) | 3.68 (1.98) | 2.35 (1.58) |
| | MD2 | 3.74 (2.06) | 2.82 (2.34) | 3.28 (2.06) | 2.34 (1.57) |
| | MD4 | 3.52 (1.92) | 2.45 (2.11) | 3.47 (2.08) | 2.21 (1.67) |
| | Motorized Spindle | 3.97 (2.11) | 2.23 (1.63) | 3.86 (1.94) | 2.52 (1.68) |
| | | | | | |
| Ports | Airport | 4.57 (1.61) | 3.36 (1.76) | 4.24 (1.66) | 3.53 (1.71) |
| | Car Terminal | 3.18 (2.32) | 2.32 (2.04) | 3.26 (1.75) | 2.99 (1.74) |
| | Cargo | 3.65 (1.85) | 4.41 (1.64) | 3.82 (1.40) | 3.17 (1.62) |

| | | | | | |
|---|---|---|---|---|---|
| | Train | 4.19 (2.02) | 3.32 (0.90) | 3.69 (1.66) | 2.90 (1.59) |
| | Ship Terminal | 3.60 (2.09) | 3.77 (1.86) | 3.66 (1.58) | 2.66 (1.57) |
| | | | | | |
| Space | Astrobotina Part 1 Space 1 | 5.11 (1.98) | 4.41 (2.80) | 5.18 (1.72) | 4.90 (2.11) |
| | Fibonacci Spiral in Nature | 5.20 (1.83) | 4.36 (2.68) | 5.05 (1.63) | 5.04 (1.66) |
| | Space 3 (Photos of the Universe) | 5.33 (1.82) | 5.23 (2.20) | 5.14 (1.46) | 5.32 (1.58) |
| | Helix Nebula | 5.24 (2.08) | 4.64 (2.03) | 5.46 (1.40) | 5.42 (1.73) |
| | Space 5 (Big Bang Theory Universe) | 4.95 (1.90) | 4.59 (2.43) | 5.14 (1.52) | 5.15 (1.71) |
