# Supplementary material for: Circumscribed interests in adolescents with Autism Spectrum Disorder: A look beyond trains, planes, and clocks
Source: PLoS One. 2017 Nov 2;12(11):e0187414. doi: 10.1371/journal.pone.0187414 (PMC5667845; doi:10.1371/journal.pone.0187414)
Supplement: S3 Table — Possible scores ranged from 1–7. (PDF) [file pone.0187414.s003.pdf]

S3 Table. Ratings for money images. Possible scores ranged from 1-7.

	Images (CDN $)	ASD Males	ASD Females	TD Males	TD Females
Money	$5	5.62 (1.62)	4.05 (1.86)	5.30 (1.45)	4.63 (1.69)
	$10	5.85 (1.52)	4.45 (2.08)	5.37 (1.59)	4.98 (1.54)
	$20	5.86 (1.62)	4.82 (2.03)	5.55 (1.45)	5.31 (1.49)
	$50	6.04 (1.41)	4.64 (2.12)	5.74 (1.52)	5.33 (1.65)
	Two dollar coin	5.54 (1.29)	4.41 (2.02)	5.18 (1.42)	5.02 (1.31)
