# Supplementary material for: Circumscribed interests in adolescents with Autism Spectrum Disorder: A look beyond trains, planes, and clocks
Source: PLoS One. 2017 Nov 2;12(11):e0187414. doi: 10.1371/journal.pone.0187414 (PMC5667845; doi:10.1371/journal.pone.0187414)
Supplement: S5 Table — (PDF) [file pone.0187414.s005.pdf]

S5 Table. Viewing times (in seconds) for High Autism Interest (HAI) images

Category	Images	ASD Males	ASD Females	TD Males	TD Females
Animations	Bugs Bunny	3.54 (2.30)	2.89 (1.94)	2.75 (1.82)	2.41 (1.50)
	Buzz Lightyear	3.29 (2.18)	2.43 (1.17)	2.90 (2.31)	2.38 (2.03)
	Homer Simpson	3.23 (1.65)	2.55 (0.87)	2.76 (1.78)	2.59 (1.87)
	Pikachu	2.88 (1.55)	2.08 (0.90)	2.23 (1.50)	2.17 (1.54)
	Scooby Doo	3.23 (2.27)	1.78 (1.25)	2.00 (1.66)	2.44 (2.74)
Simple Foods	Cereal	3.51 (2.04)	3.16 (2.09)	2.72 (2.14)	2.21 (1.46)
	Chicken	3.69 (2.92)	2.09 (0.58)	3.02 (2.82)	2.68 (2.63)
	Fries	3.63 (3.18)	3.27 (3.48)	2.62 (2.17)	2.61 (2.71)
	Ice Cream	2.82 (2.04)	1.97 (0.87)	2.23 (2.41)	2.01 (1.87)
	Toast	2.99 (1.69)	2.12 (1.32)	2.31 (1.53)	2.23 (1.71)
Gadgets	Chair	6.01 (2.88)	4.37 (3.65)	4.87 (2.63)	4.19 (2.89)
	Car	5.45 (3.10)	3.54 (1.82)	4.23 (2.62)	3.80 (2.05)
	Electronic Bike	4.64 (2.93)	3.10 (2.30)	4.32 (3.32)	3.44 (2.37)
	Headphones	4.11 (2.43)	3.19 (2.34)	2.44 (1.53)	2.65 (2.26)
	Solar Panel Sunglasses	3.94 (2.93)	3.13 (2.93)	3.34 (2.89)	2.30 (1.92)
Lego	Brick Avengers	4.13 (2.68)	4.98 (2.97)	3.47 (1.70)	3.61 (2.49)
	Art	4.12 (3.14)	2.70 (1.85)	3.45 (2.87)	2.73 (2.59)
	Polar Bear	3.93 (2.96)	3.09 (3.05)	2.80 (2.29)	2.64 (2.13)
	R2D2	3.74 (1.95)	2.70 (2.08)	2.70 (2.02)	2.24 (1.72)
	Ship	3.48 (2.40)	2.43 (1.50)	2.93 (2.54)	2.31 (2.07)
Machines	Bonsack	4.38 (1.87)	3.00 (2.01)	4.43 (1.67)	2.98 (1.61)
	MD1	3.61 (2.00)	2.82(1.95)	3.68 (1.98)	2.35 (1.58)
	MD2	3.74 (2.06)	2.82 (2.34)	3.28 (2.06)	2.34 (1.57)
	MD4	3.52 (1.92)	2.45 (2.11)	3.47 (2.08)	2.21 (1.67)
	Motorized Spindle	3.97 (2.11)	2.23 (1.63)	3.86 (1.94)	2.52 (1.68)
Ports	Airport	4.69 (3.12)	3.36 (1.93)	3.82 (2.64)	3.17 (2.41)
	Car Terminal	4.83 (2.98)	3.28 (1.54)	3.62 (2.04)	3.75 (2.69)
	Cargo	3.62 (2.45)	3.42 (2.84)	3.34 (2.42)	3.10 (2.42)

	Train	3.38 (2.06)	3.72 (3.02)	2.67 (2.02)	2.26 (1.67)
	Ship Terminal	3.57 (2.68)	2.74 (1.85)	2.63 (1.93)	2.21 (1.64)
Space	Astrobotina Part 1 Space 1	4.62 (2.83)	3.21 (1.74)	2.97 (2.46)	2.83 (1.73)
	Fibonacci Spiral in Nature	3.51 (2.43)	2.27 (1.65)	2.51 (1.93)	2.22 (1.84)
	Space 3 (Photos of the Universe)	4.00 (2.30)	2.36 (0.77)	2.64 (1.32)	2.73 (1.82)
	Helix Nebula	3.24 (2.55)	2.09 (1.54)	2.61 (1.99)	2.11 (2.15)
	Space 5 (Big Bang Theory Universe)	3.09 (2.49)	2.04 (1.33)	2.14 (1.51)	2.17 (1.93)
