# Supplementary material for: Circumscribed interests in adolescents with Autism Spectrum Disorder: A look beyond trains, planes, and clocks
Source: PLoS One. 2017 Nov 2;12(11):e0187414. doi: 10.1371/journal.pone.0187414 (PMC5667845; doi:10.1371/journal.pone.0187414)
Supplement: S6 Table — (PDF) [file pone.0187414.s006.pdf]

S6 Table. Viewing times (in seconds) for money images

	Images (CDN $)	ASD Males	ASD Females	TD Males	TD Females
Money	$5	3.95 (2.23)	4.70 (4.10)	3.34 (2.57)	3.47 (2.25)
	$10	3.55 (2.73)	2.52 (1.32)	2.60 (2.20)	2.27 (1.25)
	$20	3.32 (2.17)	3.62 (4.48)	2.56 (1.82)	2.20 (1.68)
	$50	3.58 (1.63)	3.10 (2.75)	2.64 (1.65)	2.55 (1.64)
	Two dollar coin	2.56 (1.48)	2.28 (2.02)	2.11 (1.47)	2.36 (2.13)
